# Amyloid Beta Aggregation in the Presence of Temperature-Sensitive Polymers

**DOI:** 10.3390/polym8050178

**Published:** 2016-05-02

**Authors:** Sebastian Funtan, Zhanna Evgrafova, Juliane Adler, Daniel Huster, Wolfgang H. Binder

**Affiliations:** 1 Faculty of Natural Science II, Martin-Luther University Halle-Wittenberg, Von-Danckelmann-Platz 4, D-06120 Halle (Saale), Germany; sebastian.funtan@chemie.uni-halle.de (S.F.); zhanna.evgrafova@chemie.uni-halle.de (Z.E.); 2 Institute for Medical Physics and Biophysics, Leipzig University, Härtelstraße 16-18, D-04107 Leipzig, Germany; juliane.adler@medizin.uni-leipzig.de (J.A.); daniel.huster@medizin.uni-leipzig.de (D.H.)

**Keywords:** amyloid β, fibrillation, lower critical solution temperature, thermoresponsive polymer

## Abstract

The formation of amyloid fibrils is considered to be one of the main causes for many neurodegenerative diseases, such as Alzheimer’s, Parkinson’s or Huntington’s disease. Current knowledge suggests that amyloid-aggregation represents a nucleation-dependent aggregation process *in vitro*, where a sigmoidal growth phase follows an induction period. Here, we studied the fibrillation of amyloid β 1-40 (Aβ_40_) in the presence of thermoresponsive polymers, expected to alter the Aβ_40_ fibrillation kinetics due to their lower critical solution behavior. To probe the influence of molecular weight and the end groups of the polymer on its lower critical solution temperature (LCST), also considering its concentration dependence in the presence of buffer-salts needed for the aggregation studies of the amyloids, poly(oxazolines) (POx) with LCSTs ranging from 14.2–49.8 °C and poly(methoxy di(ethylene glycol)acrylates) with LCSTs ranging from 34.4–52.7 °C were synthesized. The two different polymers allowed the comparison of the influence of different molecular structures onto the fibrillation process. Mixtures of Aβ_40_ with these polymers in varying concentrations were studied via time-dependent measurements of the thioflavin T (ThT) fluorescence. The studies revealed that amyloid fibrillation was accelerated in, accompanied by an extension of the lag phase of Aβ_40_ fibrillation from 18.3 h in the absence to 19.3 h in the presence of the poly(methoxy di(ethylene glycol)acrylate) (3600 g/mol).

## 1. Introduction

A wide range of neurodegenerative diseases, such as Alzheimer’s, Parkinson’s or Huntington’s disease, is related to misfolding and aggregation of particular amyloid proteins, leading to the formation of insoluble fibrils [1]. Despite the fact that native soluble proteins associated with these illnesses have very different amino acid sequences and, therefore, different structures, the resulting insoluble aggregates are mainly straight, unbranched and share a common β-sheet secondary structure [2,3,4,5,6,7]. The fibrillation kinetics of the amyloid protein has been broadly studied and can be described by a sigmoidal curve featuring three characteristic regions known as the lag phase, the growth phase and the saturation phase or “plateau region” [3,4,8].

**Figure 1:**
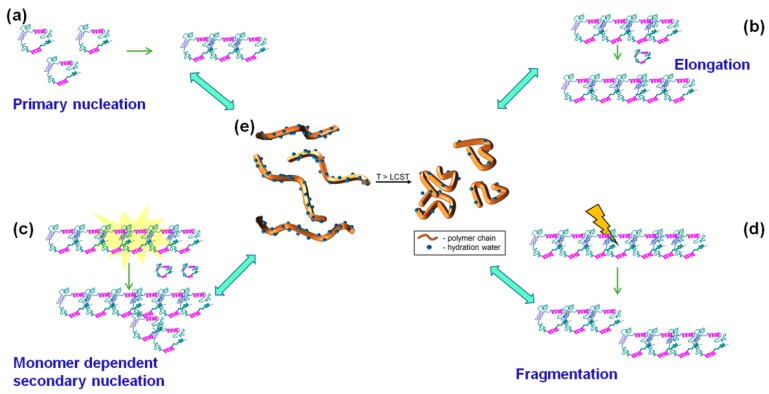
Molecular events during the lag time (**a**–**d**): (**a**) primary nucleation; (**b**) elongation; (**c**) monomer-dependent secondary nucleation; (**d**) fragmentation creates new elongation sites in the system; and (**e**) transition of a thermoresponsive polymer from the hydrated coil to the collapsed state. Prior to heating, the polymer chain (brown) is more hydrated. When the cloud point temperature (*T*_CP_) has been reached, the chain collapses to a compact globule by releasing most of the hydration water (blue), and further aggregation of the collapsed chains is possible [3,12,13,14,15,16]. LCST: lower critical solution temperature.

At the very early stage of the lag phase, when no (larger, detectable) aggregates are present, native soluble proteins combine to form primary nuclei, which are referred to as oligomeric species and protofibrils characterized by a significant β-sheet conformation (Figure 1a) [9,10]. These primary nuclei then elongate (Figure 1b), and a secondary nucleation catalyzed by the surface of a growing filament takes place (Figure 1c). Fragmentation (Figure 1d) as a consequence of external forces (e.g., mixing or shaking), which will generate new active chain ends, is also possible and will accelerate fibril growth [11]. Although the fraction of the aggregated fibrils during the lag phase is relatively low and difficult to characterize, experimental proof confirms that the mature fibrils are already present in the system [3,12,13,14,15]. The second phase of the amyloid protein fibrillation is characterized via a growth phase, which is the thermodynamically-favorable addition of monomers to the growing prefibrillar structures, [3,8,14,15], followed by the last step, the saturation phase, where the concentration of the mature fibril is very high and the concentration of the monomeric species has achieved a constant value [3,8,14,15].

Beside small molecules [17,18] and proteins [19,20,21,22], especially polymers are promising candidates for the inhibition of the fibrillation as has been shown for the poly-(l)-lysine (PLL) [23], dendrimers [11,24,25] and polymeric nanoparticles [26,27,28]. Thus, the presence of bilayer membranes [3,29], micellar aggregates [3,30,31,32,33] and nanoparticles [27,34,35] has shown to significantly alter the aggregation process, where especially the interplay between Aβ with artificial membranes is often seen as an important factor of the Aβ fibrillation *in vivo* [3]. *In vitro* aggregation of the amyloid protein can be delayed by means of interaction with, e.g., phospholipid membranes, leading to a reduction of the amyloid-formation from initially 2–9 h in the presence of liposomes made from dioleoyl-phosphatidylcholine (DOPC) in the gel phase [29]. Furthermore, surfactant micelles interfere with the formation of the Aβ fibrils in a concentration-dependent manner [3,30,31,32,33], as in the presence of sodium dodecyl sulfate (SDS) the lag time for a solution containing 150 mM Aβ_40_ is decreasing from 7.5 ± 2.0–1.9 ± 0.2 h at 0.9 mM SDS and 1.2 ± 0.2 h at 2.75 mM SDS (below its critical micelle concentration (cmc)), whereas a higher concentration of SDS (12.5 mM, above the cmc) suppressed fibrillation [33]. The same effect was observed for Aβ-nanoparticle systems [27,34,35], where the presence of 0.05 or 1.1 mg/mL of amine-modified polystyrene nanoparticles in a 16 mM Aβ system changed the lag time from 138 ± 20 min to around 50 and 500 min, respectively, also reporting the formation of stickier and shorter fibrils [27]. However, contradictory results have been achieved with regard to the interference of polymers and polyelectrolytes with Aβ fibrillation kinetics [36].

To the best of our knowledge, the influence of thermoresponsive polymers on the fibrillation pathways of amyloids has not yet been investigated. The current study provides a first report on the influence thermoresponsive polymers exert on the fibrillation of an amyloid protein, mixed noncovalently into a fibrillating amyloid system. An important requirement for such an experiment is the correct choice of the lower critical solution temperature (LCST) behavior of the polymer, which should be chosen close to the temperature, where amyloid aggregation is observed (experimentally *in vitro*). As the LCST is also referred to as the cloud point temperature (*T*_CP_), where the conformation changes from a hydrated coil to a collapsed globule by the release of most of the hydration water (coil-globule transition; see Figure 1e) [16], it is tempting to speculate about the effects of such a transition on the amyloid aggregation, most of all with respect to the macroscopic kinetic aggregation behavior.

Besides the well-known poly(*N*-isopropylacrylamide) (PNIPAM) [37,38,39], especially poly(oxazolines) (POx) [40,41,42,43] and polyacrylates containing a PEG part in the repeating unit [44,45] are widely-used thermoresponsive polymers, allowing one to tune the LCST behavior by variation of the nature and amount of the used (co)-monomers: hydrophilic monomers in the chain shift the LCST to higher values, whereas the incorporation of hydrophobic monomers will decrease the LCST [42,45]. Thus, hydrophilic PEtOx displays an LCST of *T*_CP_ = 60–68 °C [40], whereas poly(2-isopropyl-2-oxazoline) (P*i*PrOx) shows an LCST around body temperature of *T*_CP_ = 36–39 °C [41]. Nearly any value for the LCST can be adjusted, e.g., by copolymerization (P(*n*PrOx-EtOx) [42,46], P(*n*PrOx-*i*PrOx) [42], P(*i*PrOx-*n*BuOx) [47], P(*i*PrOx-NonOx) [47], P(c-PropOx-EtOx) [48]) or by adjusting the polymer concentration [40], the molecular weight [40,46] or the end groups [49,50]. Similarly, thermoresponsive polyacrylates, containing a PEG part in the repeating unit [44,45], display an LCST behavior [45,51]. It was found that the cloud point can be adjusted steplessly between 9 and 90 °C.

Here, we describe the design and syntheses of two different types of polymers (poly(oxazolines) and poly(methoxy di(ethylene glycol)acrylates)), both showing an LCST at temperatures close to the *in vitro*-aggregation temperature of amyloids. Subsequently, we study their effect on amyloid aggregation probed by conventional aggregation assays. A special focus was placed on the comparison of the two different hydrophilic polymers.

## 2. Materials and Methods

### 2.1. Materials

The following chemicals were purchased from Sigma Aldrich (Taufkirchen, Germany): 2,2′-azobis(2-methylpropionitrile) (AIBN), ethanolamine, deuterated chloroform, isobutyronitrile, methyl trifluoromethanesulfonate, *N*-methylpropargylamine, THF (HPLC grade), valeronitrile and zinc acetate dihydrate. *N*,*N*-diethylamine and propargyl tosylate were purchased from Fluka (Taufkirchen, Germany). Calcium hydride was bought from Alfa Aesar (Karlsruhe, Germany). Sodium chloride and sodium sulfate were bought from Roth (Karlsruhe, Germany). Ce(SO_4_)·4H_2_O and (NH_4_)_6_Mo_7_O_24_·4H_2_O were obtained from VEB (Eschborn, Germany). Concentrated sulfuric acid was purchased from Th. Geyer (Renningen, Germany). Deuterated chloroform was obtained from Chemotrade (Düsseldorf, Germany), and DMF (HPLC grade) was purchased from VWR-Prolabo (Darmstadt, Germany).

Aβ_40_ peptide was synthesized using the standard F-moc solid phase synthesis strategy at the core unit “Peptid-Technologien” of the Medical Faculty of the University of Leipzig [52].

The oxazoline monomers were stored over calcium hydride and were freshly distilled before usage. All solvents were freshly distilled and degassed by bubbling with nitrogen for at least 20 min prior to usage. Acetonitrile (ACN) was dried with calcium hydride by boiling for several hours followed by distillation. AIBN was recrystallized from ethanol twice before usage.

### 2.2. Methods

^1^H-NMR spectra were recorded on a Varian Gemini 2000 (400 MHz) (Agilent, Waldbronn, Germany) or on a Varian Unity Inova 500 (500 MHz) (Agilent, Waldbronn, Germany) using MestReNova software (Version 6.0.2-5475) (Mestrelab Research, Santiago de Compostela, Spain) for the evaluation of the results. NMR spectra were measured at 27 °C using deuterated chloroform (CDCl_3_). All chemical shifts (δ) were given in parts per million (ppm) relative to trimethylsilane (TMS) and referred to the solvent signal (CDCl_3_: 7.26 ppm (^1^H), 77.0 ppm (^13^C)).

MALDI-TOF-MS analysis was carried out using a Autoflex III Smartbeam (Bruker Daltonics, Bremen, Germany) equipped with a nitrogen laser (337 nm) working in linear and reflection modes. The obtained data were evaluated using flexAnalysis software (Version 3.0) (Bruker Daltonics, Bremen, Germany). The matrix solution was prepared by dissolving 1,8,9-anthracenetriol (dithranol) in THF at a concentration of 20 mg/mL. The polymer was dissolved in THF (10 mg/mL) and mixed with sodium trifluoroacetate (10 mg/mL in THF). The ratio between the matrix, the analyte and the salt was 100:10:1.

Gel permeation chromatography (GPC) for the poly(methoxy di(ethylene glycol)acrylates) was performed on a Viscotek GPCmax VE 2002 (Malvern, Crowthorne, UK) using a H_HR_-H Guard-17360 precolumn and a GMH_HR_-*N*-18055 column with THF as the solvent and a VE 3580 IR detector for refractive index determination. A polystyrene standard (*M*_P_ = 1000–115,000 g/mol) was used for external calibration. Column and detector temperatures were held at 22 and 35 °C, respectively, and the flow rate was set to 1 mL/min. The concentration of all samples was 3 mg/mL.

GPC measurements of the POx were performed on a Viscotek GPCmax VE 2001 (Malvern, Crowthorne, UK using a H_HR_-H Guard-17369 and a GMH_HR_-N-18055 column with DMF as the eluent at 60 °C and via detection of the refractive index with a VE 3580 RI detector from Viscotek at 35 °C. The external calibration was done using a polystyrene standard (*M*_P_ = 1000–115,000 g/mol). The concentration of all samples was 5 mg/mL, and the flow rate was 1 mL/min.

Turbidimetry measurements were done using the UV–Vis spectrometer HP 8543 (Hewlett-Packard, Waldbronn, Germany). By coupling with a Peltier element HP 89090A (Hewlett-Packard, Waldbronn, Germany), controlled heating with a heating rate of 0.5 °C/min was possible. The observed wavelength was λ = 500 nm. For all measurements in water, a 1 wt % solution of the polymer was used. Measurements in buffers were done as follows: for the poly(methoxy di(ethylene glycol)acrylates), sodium phosphate buffer (25 mmol/L), containing sodium chloride (150 mmol/L) at pH = 9.2, was used. *T*_CP_ was detected at 50% of transmission. The POx were measured in sodium borate buffer (50 mmol/L, pH = 9.0) as 1 wt % solutions. Only the P(*i*PrOx-*n*BuOx) copolymers were measured as 0.25 wt % solutions due to their poor solubility. *T*_CP_ was chosen as the onset temperature, when the first drop of the transmission was observed.

Fluorescence measurements for the fibrillation kinetics of the Aβ_40_ peptide in the absence and in the presence of the polymers were measured on a Tecan infinite M200 microplate reader (Tecan Group AG, Männedorf, Switzerland). Data were analyzed using standard protocols from the literature [53]. Thioflavin T (ThT) was used as a fibril indicator, because it shows increasing fluorescence intensity at λ = 482 nm by binding to β-sheet-rich structures, such as the formed Aβ_40_ fibrils [54]. Measurements were performed in a 96-well plate. Mixtures of Aβ_40_ and the polymers were dissolved in sodium phosphate buffer (25 mmol/L) at pH 9.2, containing sodium chloride (150 mmol/L), ThT (20 μmol/L) and 0.01% NaN_3_ to prevent bacterial growth. The used concentrations were: Aβ_40_: 230 μM; polymer **3a**: 196 μM; polymer **3b**: 113 μM; polymer **3c**: 260 μM; and polymer **6c**: 230 μM. One hundred fifty microliters of each solution were pipetted into the wells. The temperature was 37 °C; the excitation wavelength was set to 450 nm; and the emission was measured at 485 nm every 30 min for at least three days. Two different shaking protocols were used: 30 min cycles of 5 min shaking (at a 1-mm shaking amplitude), 5 min waiting, 5 min shaking followed by the measurement (Protocol I) and 25 min and subsequent 10 s shaking followed by the measurement (Protocol II). The fluorescence intensity was fitted using the following equation [53]:

(1) $$I=F_{1}+m_{1}t+\;\frac{F_{2}+m_{2}t}{1+e^{-\left[\left(t-t_{char}\right)/\tau \right]}}$$

where *t*_char_ is the characteristic time, where the fluorescence intensity reaches half its maximum and the lag time is given by *t*_char_-2τ with τ as the inverse of the rate constant [53].

Thin-layer chromatography (TLC) was performed using “Merck silica gel 60” plates (Merck, Darmstadt, Germany). Spots on the TLC plate were visualized using oxidizing agent “blue” stain or UV light (254 or 366 nm). “Blue” stain was prepared as follows: (NH_4_)_6_Mo_7_O_24_∙4H_2_O (1 g) and Ce(SO_4_)_2_∙4H_2_O (1 g) were dissolved in a mixture of distilled water (90 mL) and concentrated sulfuric acid (6 mL). Subsequent column chromatography was carried out using high purity-grade Merck 60 (230–400 mesh particle size) silica gel (Darmstadt, Germany).

### 2.3. General Procedure for the Syntheses of the Poly(methoxy di(ethylene glycol)acrylates) ***3***

As an example, the synthesis for a projected molecular weight of *M*_n_ = 3800 g/mol is described. The reaction was carried out using a round-bottomed flask equipped with a magnetic stirrer, a rubber septum and a balloon filled with argon. Prior to the reaction, a mixture of monomer **1** (242.60 mg, 234.80 μL, 1.39 mmol), chain transfer agent (CTA) **2** (15.10 mg, 0.063 mmol), AIBN (1.04 mg, 0.0063 mmol) and DMF (0.34 mL) was bubbled with argon for 25 min and subsequently placed into a preheated oil bath at 70 °C. The reaction was stirred for six hours before it was opened to air and cooled by means of an ice bath. The resulting bright yellow polymer was precipitated into *n*-hexane (3 × 70 mL) and dried in a high vacuum within two days. The polymeric product **3** was characterized via size exclusion chromatography (SEC), matrix-assisted laser desorption/ionization mass spectrometry (MALDI-TOF MS) (for isotopic pattern simulation see Supplementary Materials, Figure S1), ^1^H-NMR (see Supplementary Materials, Figure S2) and turbidimetry (Table 1). Thus, the expected final polymer was obtained as truly proven via these experimental methods.

^1^H-NMR (400 MHz, CDCl_3_): *δ* = ppm 4.83 (m, 1H_a_, –C*H*), 4.19 (s, 42H_e_, –C*H*_2_), 3.62 (m, 84H_f_,_g_, –C*H*_2_), 3.53 (m, 42H_h_, –C*H*_2_), 3.36 (m, 63H_i_, –C*H*_3_), 2.34 (s, 21H_d_, –C*H*), 1.92–1.41(m, 46H_c_,_k_,_l_, –C*H*_2_), 1.15 (m, 3H_b_, –C*H*_3_), 0.93 (t, 3H_m_, –C*H*_3_).

### 2.4. Syntheses of the Poly(oxazolines) ***6**–**9***

Similar to known procedures [47,55], 2-isopropyl-2-oxazoline **4** (*i*PrOx) and 2-*n*-butyl-2-oxazoline **5** (*n*BuOx), served as the monomers [56] for the cationic ring-opening polymerization for the POx. Polymers **6**, **7** and **9** were all initiated with propargyl tosylate, but polymers **6** and **9** were quenched with water, whereas polymer **7** was quenched with *N*,*N*-diethylamine. The synthesis of polymer **8** was initiated with methyl trifluoromethanesulfonate and quenched with *N*-methylpropargylamine. Results for the characterization and the measured LCSTs for all polymers are given in Table 1 (turbidimetry, molecular weights and polydispersities). Detailed procedures for all reactions and the corresponding characterizations are given in the Supplementary Materials.

### 2.5. General Procedure for the Syntheses of the Poly(oxazolines) ***6**–**8***

2-Isopropyl-2-oxazoline (2.00 g, 2.10 mL, 17.63 mmol), dry ACN (8.81 mL) and the appropriate amount of initiator (propargyl tosylate for polymers **6** and **7**, methyl trifluoromethanesulfonate for polymer **8**) were added to a Schlenk tube, which was subsequently sealed with a rubber septum. The mixture was stirred at room temperature for one hour and consecutively for 48 h at 80 °C. Living chain ends were quenched by the addition of the respective quencher (**6**: water; **7**: *N*,*N*-diethylamine; **8**: *N*-methylpropargylamine) and further stirring for 24 h at 60 °C. After evaporation of the solvent, the residue was dissolved in dichloromethane DCM (5.0 mL) and was extracted with water (5 × 30.0 mL). The combined aqueous phases were back extracted using DCM (10 × 30.0 mL). Subsequently, the organic phases were combined and dried over sodium sulfate. After filtration, most of the solvent was removed, and the remaining viscous solution was precipitated three times in a cold mixture of diethyl ether/*n*-hexane (1:1) to obtain the pure polymer. A representative ^1^H-NMR spectrum is given in the Supplementary Materials for polymers **6**, **7** and **8** (Figures S3–S5).

^1^H-NMR (400 MHz, CDCl_3_, 27 °C): *δ* (ppm) = 4.09 (s, 2H, *H*_4_), 3.76–3.26 (m, C*H*_2_ of the repetitive unit), 3.01–2.55 (m, C*H* of the repetitive unit), 1.09 (s, C*H*_3_ of the repetitive unit).

### 2.6. General Procedure for the Syntheses of Poly(2-isopropyl-2-oxazoline-grad-2-n-butyl-2-oxazoline) ***9***

The procedure was done as described for the homopolymerization of 2-isopropyl-oxazoline. A mixture of 2-isopropyl-2-oxazoline (0.54 mL, 0.51 g, 4.50 mmol), 2-*n*-butyl-oxazoline (63.60 mg, 0.50 mmol), propargyl tosylate (50.86 μL, 61.80 mg, 0.29 mmol) and ACN (2.50 mL) was stirred for one hour at room temperature in a Schlenk tube. After stirring for 48 h at 80 °C, the reaction was quenched by the addition of water (20.90 μL, 20.90 mg, 1.16 mmol). The reaction was stirred for a further 24 h at 60 °C, and the work-up was done as described for the poly(2-isopropyl-2-oxazoline). A representative ^1^H-NMR spectrum is given in the Supplementary Materials (Figure S6), as well as a table with full characterization of the synthesized copolymers (initial monomer ratio, molecular weight as determined from ^1^H-NMR and GPC, polydispersity index (PDI) and copolymer composition as determined from ^1^H-NMR) (Table S1).

^1^H-NMR (400 MHz, CDCl_3_, 27 °C): *δ* (ppm) = 4.03 (s, 2H, *H*_9_), 3.50–3.25 (m, C*H*_2_ of the repetitive unit, *H*_1_ + *H*_4_), 2.90–2.50 (m, C*H* of the repetitive unit), 2.32–2.15 (m, C*H*_2_ of the repetitive unit, *H*_5_), 1.50 (s, C*H*_2_ of the repetitive unit, *H_6_),* 1.25 (s, C*H*_2_ of the repetitive unit, *H*_7_), 1.02 (s, C*H*_3_ of the repetitive unit, *H*_3_), 0.82 (s, C*H*_3_ of the repetitive unit, *H*_8_).

## 3. Results and Discussion

### 3.1. Syntheses of the Polymers

**Scheme 1:**
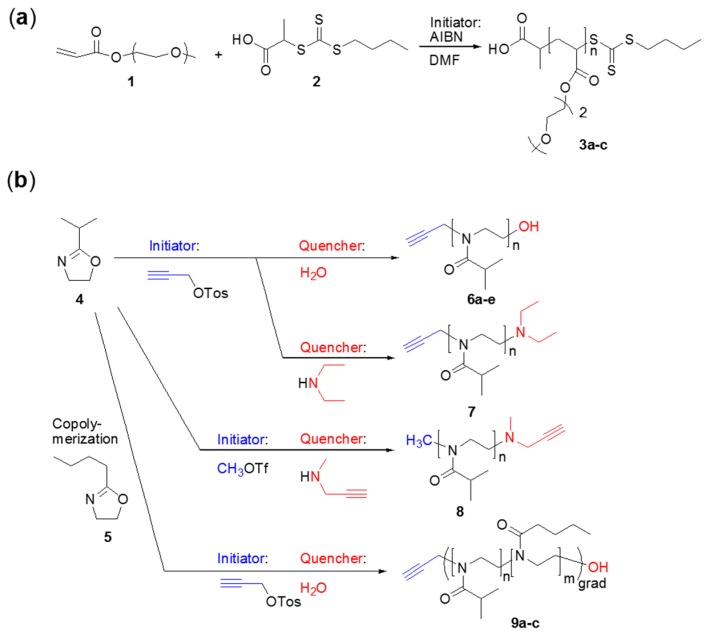
Synthetic pathway for the thermoresponsive polymers that were used for the LCST and fibrillation measurements: (**a**) preparation of poly(methoxy di(ethylene glycol)acrylates) **3**; (**b**) preparation of the poly(2-isopropyl-2-oxazoline) homopolymers **6**–**8** (using different initiation and quenching methods) and the poly(2-isopropyl-2-oxazoline-*grad*-2-*n*-butyl-2-oxazoline) copolymers **9** (*n* = 9–11; *m* = 3–6).

We synthesized two different polymers, both displaying an LCST (see Scheme 1a,b): on the one hand, poly(methoxy di(ethylene glycol)acrylates) **3a**–**c** were prepared by reversible addition-fragmentation chain-transfer polymerization (RAFT) [57,58] starting from methoxy di(ethylene glycol)acrylate **1** [59] and a chain transfer agent (CTA) (2-(*n*-butyltrithiocarbonylthio) propionic acid **2** [60]) with AIBN as the initiator. The molar ratio between monomer:CTA:AIBN (**3a**: 22:1:0.1; **3b**: 50:1:0.1; **3c**: 90:1:0.1) was used to adjust the intended molecular weight, generating polymers with an LCST varying from ~34–45 °C, whereas the poly(oxazolines) **6**–**9** where designed as homo- and co-polymers, thus addressing a wide range of different LCSTs and molecular weights via living cationic ring-opening polymerization.

**Figure 2:**
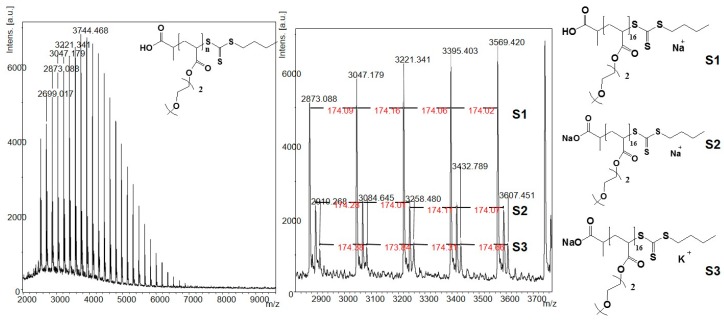
MALDI-TOF spectrum of poly(methoxy di(ethylene glycol)acrylate) (**3a**). S1, Series 1.

As a representative example, the MALDI-TOF of polymer **3a** is shown in Figure 2, displaying three different series (Series S1–S3), corresponding to the repeating unit (difference ~174 Da) and different types of attached ions. The first series at 3047.179 Da can be assigned to poly(methoxy di(ethylene glycol)acrylate) with a formula of [HOOCC_2_H_4_(C_8_H_14_O_4_)_16_S_3_C_5_H_9_]Na^+^. The main signal of the multiplet chosen for Series S2, which appears at 3069.360 Da, can be assigned to [NaOOCC_2_H_4_(C_8_H_14_O_4_)_16_S_3_C_5_H_9_]Na^+^, whereas the minor Series S3 appears at 3084.645 Da and is assigned to the polymer with the formula of [NaOOCC_2_H_4_(C_8_H_14_O_4_)_16_S_3_C_5_H_9_]K^+^. For simulation of the isotopic pattern, check the Supplementary Materials (Figure S1).

Further characterization via NMR spectroscopy is shown in the Supplementary Materials (Figure S2), finally proving both the end groups and the true structure of polymer **3**. Similarly, the poly(oxazolines) **6**–**9** were characterized via NMR spectroscopy and GPC (see Supplementary Materials Figures S3–S6).

### 3.2. Turbidimetry of the Polymers ***3**, **6**–**9***

In preparation of the subsequent fibrillation experiments (amyloid aggregation in the presence of polymers), first-hand experimental information on the LCST influence of the molecular weight and the addition of salts (buffer solutions are required for respective fibrillation conditions), the concentration of the polymer via appropriate extensive turbidimetric measurements was accomplished. First experiments were done with P*i*PrOx (**6**, **7** and **8**), whose LCST behavior is well documented in the literature [41,42,47,50], followed by poly(methoxy diethylene glycol)acrylate, which is less intensely studied in the literature [45,61,62]. The results of the turbidimetric measurements are given in Table 1.

**Figure 3:**
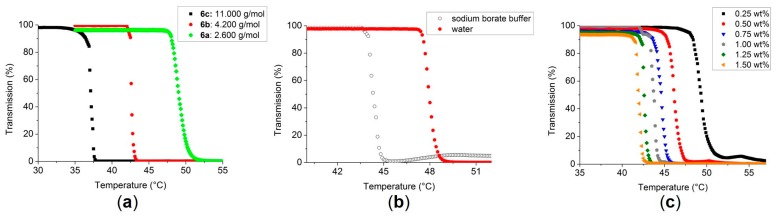
LCST measurements for the POx as 1 wt % solutions: (**a**) influence of the molecular weight for the polymers **6a**, **6b** and **6c**; with an increasing molecular weight, the LCST is decreasing; (**b**) sodium borate (50 mmol/L, pH = 9.0) decreases the LCST of P*i*PrOx **6a** (2600 g/mol) by nearly 4 K from 47.5 down to 43.6 °C; the curve with the filled symbols (red) refers to a measurement in water, whereas the curve with the open symbols (black) refers to a measurement in buffer; (**c**) with an increasing concentration, the LCST for polymer **7** (3100 g/mol) is shifted to lower values and can be tuned between 48.2 °C (0.25 wt %) and 41.3 °C (1.50 wt %) within the tested concentration range.

For the P*i*PrOx polymers (**6**, **7** and **8**), the same influence of the molecular weight on the LCST was found as described in the literature [46,55,63]. Thus, an increase in the molecular weight led to a decrease of the LCST. By varying the molecular weight, it was possible to tune the LCST in a range of ±5 K. P*i*PrOx **6a** with a molecular weight of 2600 g/mol (GPC) exhibits an LCST of 47.5 °C (in H_2_O), whereas P*i*PrOx **6c** with a molecular weight of 11,000 g/mol (GPC) displays an LCST of 36.4 °C (in H_2_O). The measured curves of some of the P*i*PrOx are shown in Figure 3a and illustrate the influence of the molecular weight on the LCST.

A significant influence of the used buffer on the LCST was observed when changing to sodium borate buffer as the solvent (required for the amyloid fibrillation assays; 50 mmol/L, pH = 9.0), as the presence of the sodium borate provoked a decrease of the LCST in the range of 1–4 °C, except for polymer **8**. As the ions in the buffer are able to weaken the hydrogen bonds between the polymer chains and water molecules, the release of water is facilitated, consequently shifting the LCST to lower temperatures (salting out effect). Figure 3b exemplary shows the measured curves for the polymer **6a** in pure water and in sodium borate buffer as a comparison.

Furthermore, the concentration dependency of the LCST was measured for polymer **7** in the range from 0.25–1.5 wt % in water. The results are presented in the Supplementary Materials (Table S2), and the measured curves are shown in Figure 3c. For the lowest concentration (0.25 wt %), an LCST of 48.2 °C and for the highest concentration (1.5 wt %) an LCST of 41.3 °C were measured. Apparently, a change of the concentration by 0.25 wt % has a significant effect on the LCST, especially at lower concentrations.

As expected, the copolymerization with the more hydrophobic *n*BuOx resulted in a decrease of the LCST as described in the literature [47]. Because of the high amount of *n*BuOx from 21%–35%, the LCST was reduced to 27.2 and 15.3 °C respectively, illustrating the strong influence of the hydrophilic/hydrophobic ratio of a polymer on its LCST. By measuring the samples in sodium borate buffer, the LCST could be further decreased to 14.2 °C (see Supplementary Materials, Figure S7).

In a similar manner, LCST data were obtained via turbidimetric measurements for the polyacrylates **3** as explained in the experimental part, showing the same unexpected influence of the molecular weight on the LCST as described in the literature [59,61,62] (see Table 1). Thus, LCSTs ranging from 34.4 °C (polymer **3a**) to 45.1 °C (polymer **3c**) could be determined, demonstrating a strong increase ofthe *T*_CP_ with increasing molecular weight [62].

Upon determining the LCST in sodium phosphate buffer at pH = 9.2, keeping the polymer concentration at 230 μM, polymer **3a** does not display an LCST under the applied conditions (measurement range = 25–70 °C). Cloud point temperatures of samples **3b** and **3c** were detected at 52.7 °C (40.4 °C in water) and 49.5 °C (45.1 °C in water), indicating that the addition of ions stabilize the macromolecules in solution, however with a less pronounced effect for higher molecular weights (**3b**: ∆*T*_cp_ = 12.3 °C; **3c**: ∆*T*_cp_ = 4.4 °C) (see Supplementary Materials for details on the measured curves, Figures S8 and S9).

### 3.3. Fibrillation of Aβ_40_ in the Presence of Polymers ***6** and **3***

The choice of the polymers for the amyloid fibrillation measurements was based on the reverse thermoresponsive behavior of the POx and polyacrylates. These polymers are water and buffer soluble, and the *T*_cp_ could be adjusted over a wide range of temperatures. Polyacrylates **3a**, **3b** and **3c** (displaying molecular weights below and above the molecular weight of Aβ_40_) serve as a good selection for the Aβ aggregation studies due to the different LCSTs. P*i*PrOx **6c** was chosen due to the molecular weight of the polymers **3c** and **6c** being close to each other (14,600 g/mol and 11,000 g/mol, respectively), thus probing whether there is an influence on the fibrillation of the Aβ_40_ due to the structural differences of the polymers.

Upon addition of an LCST-type polymer to an aggregating amyloid, a number of possible scenarios can be considered. Thus, by mixing of the polymer with the Aβ protein, the polymer can either inhibit or enhance fibrillation, as observed before [36]. At *T* < *T*_LCST_ and a concentration *C*_amyloid_ < *C*_polymer_, the amyloid could evenly distribute within the polymer phase, in turn preventing the amyloid/amyloid contact formation and, thus, reducing or even eliminating fibrillation. At a concentration *C*_amyloid_ < *C*_polymer_, the polymer could also form a protective layer around the native proteins, and at *C*_amyloid_ > *C*_polymer_, the polymer might boost the fibrillation by acting as a catalytic surface. If present above its LCST, several scenarios are possible, e.g., that the polymer can hinder the fibrillation of the amyloid by evenly binding the native proteins to its surface and therefore preventing the formation of bigger oligomers or alternatively accelerating the fibrillation by acting as a seed.

#### 3.3.1. Effect of Poly(oxazolines) on Aβ_40_ Aggregation

**Figure 4:**
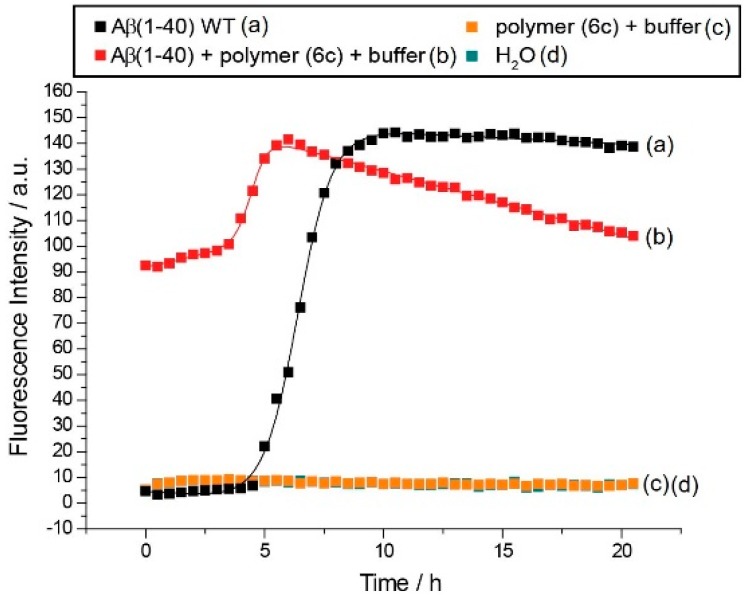
Thioflavin T fluorescence intensity of the Aβ_40_/polymer (**6c**) mixture (**b**) compared to WT Aβ_40_ (**a**) and the pure polymer (**6c**) (**c**) in buffer solution, as well as pure water (**d**). Both the characteristic and lag time are shortened for the peptide/polymer mixture compared to WT Aβ_40_ fibrillation kinetics. The characteristic time, which reports the time at which half the fluorescence maximum is reached, decreases from 6.4 down to 4.5 h, and the lag time is reduced from 4.9 down to 3.7 h. The higher initial thioflavin T (ThT) fluorescence of Aβ_40_ in the presence of **6c** (**b**) could be related to an initial nucleation that was too fast to be recorded in our experimental setup. The observed decrease of the fluorescence after *t* = 5 h (**b**) often is the result of the precipitation of the sample.

For the first fibrillation measurements, equimolar amounts of Aβ_40_ (wild-type, WT) and the corresponding polymer were dissolved in sodium phosphate buffer (25 mM, pH = 9.2, containing 150 mM NaCl) at a concentration of 230 μmol/L for both Aβ_40_ and the polymer. Tracking of the fibril growth was done by using the fluorescence dye thioflavin T (ThT) (20 μmol/L), as ThT binds to β-sheet-rich structures showing an increasing fluorescence at λ = 482 nm, which in turn allows the measurement of the kinetics of the fibril growth [54]. Measurements were done at 37 °C using a well plate, which allowed the triple measurement of each sample simultaneously (Protocol I, see Methods). The time-dependent development of the ThT at λ = 482 nm is shown in Figure 4. Upon the addition of P*i*PrOx **6c**, Aβ_40_ shows a significantly increased fluorescence (Trace b), indicative of fibril formation with a characteristic time *t*_char_ = 4.5 h in the presence of polymer **6c**. To exclude interactions between the polymer and ThT, the fluorescence of the pure P*i*PrOx in buffer solution in the absence of Aβ_40_ was measured as a control experiment (Trace c), in turn excluding nonspecific ThT/P*i*PrOx (**6c**) interactions. For a comparison, the time-dependent development for the ThT fluorescence at λ = 482 nm for WT Aβ_40_ is given in the figure (Trace a), indicating a characteristic time for fibrillation of Aβ_40_ of t_char_ = 6.4 h.

Thus, the faster fibril growth upon the addition of polymer **6c** can be attributed to a shortening of the lag phase of fibrillation. As under the used conditions the LCST of **6c** was 36.2 °C, the polymer was above its LCST and consequently precipitated during the measurement, in turn acting as a seed for the Aβ_40_ monomers and favoring oligomerization.

#### 3.3.2. Effect of Poly(methoxy di(ethylene glycol)acrylates) on Aβ_40_ Fibrillation

**Figure 5:**
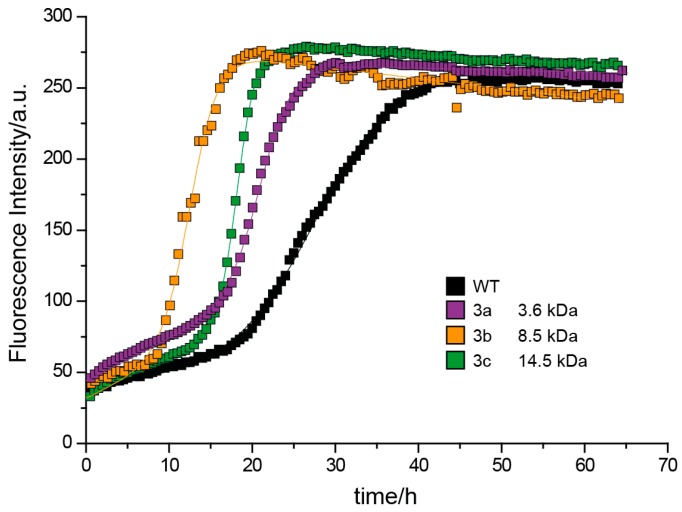
Time-dependent evolution of the ThT fluorescence intensity at λ = 482 nm of the polymer/Aβ_40_ mixtures. For polymers **3b** and **3c**, the lag phase is shortened, while for polymer **3a**, the lag phase is increased by one hour from *t* = 18.3 h (for pure Aβ_40_) to *t* = 19.3 h.

Measuring amyloid aggregation via the ThT fluorescence assay (Protocol II, see Methods) in the presence of the acrylates **3a**, **3b** and **3c** was accomplished in 230 μM Aβ_40_ buffered solutions, mixing with buffered solutions of the polymers with different chain lengths and concentrations (Figure 5). In all cases, the lag time was reduced, and the saturation phase of fibrillation was achieved more quickly. Furthermore, the slope of all three aggregation curves became steeper, in comparison to WT Aβ_40_, implying that the presence of the polymer is boosting the protein fibrillation and enhancing secondary nucleation and the elongation rates.

Remarkably, the duration of the lag phase seems to be decreased in the case of the polymers **3b** and **3c**, but slightly increased from *t* = 18.3 h to *t* = 19.3 h in the case of the polymer **3a**, indicating that polymer **3a**, having the closest molecular weight compared to the Aβ_40_ (~4300 g/mol), is able to interact with the native protein interfering with the primary nucleation process. This phenomenon is extremely interesting due to the prospective control over the Aβ_40_ fibrillation upon the change of the molecular weight or the concentration of the polymer and will be studied in detail in the upcoming publications. Nevertheless, the WT Aβ_40_ peptide shows the longest characteristic times compared to the peptide/polymer mixtures.

The summarized values for the P*i*PrOx, as well as for the poly(methoxy di(ethylene glycol)acrylates) are given in Table 2, as well as the cloud points for those polymers under the conditions that were used for the fibrillation experiments.

#### 3.3.3. Discussion of Aβ_40_ Aggregation Experiments

The influence of polymers, such as neutral polymers [36], polyelectrolytes [36], dendrimers [11,24,25] or polymeric nanoparticles [26,27,28], on amyloid aggregation has been studied. Often, retarding effects on the fibrillation of Aβ have been observed. Amyloid formation has been found to be promoted by glycosaminoglycans [64] and positively-charged polymers, such as poly(diallyldimethylammonium chloride) (PDDA), poly(ethylenimine) (PEI) and poly(lysine) hydrobromide [36]. Most relevant to our situation is the study by Assarsson *et al.*, in which the effect of charged polymers and polyamino acids on the Aβ fibril formation was studied [36]. While an accelerating effect of all positively-charged polyelectrolytes on Aβ fibril formation was found, neutral (poly(threonine) and negatively-charged polymers poly(acrylic acid sodium salt) (PAA) and poly-glutamic acid sodium salt did not interfere with the kinetics of Aβ fibrillation. In contrast, all neutral polymers investigated in our study also accelerated the fibrillation kinetics of Aβ_40_. In agreement with the aforementioned results, the lag time of Aβ fibril formation decreased, and the growth phase showed a steeper slope. This is akin to the situation where the Aβ concentration is systematically increased [65].

It should finally be mentioned that the concentration of the polymers added to the Aβ preparations is too low to influence the Aβ fibrillation by crowding through excluded volume effects. However, somehow, it is likely that the presence of the polymer leads to an increase in the local Aβ concentration, which favors nucleation of the peptide and leads to the observed increased fibrillation kinetics.

## 4. Conclusions

Amyloid aggregation was studied in the presence of hydrophilic polymers, able to display an LCST in the range between 35 and 45 °C. For this effect, we have synthetically prepared a large number of hydrophilic polymers, adjusting the LCST to 35–45 °C by either homo- or co-polymerization. Poly(oxazolines) **6**–**9**, prepared via cationic ring opening polymerization, served as one class of hydrophilic polymer, whereas poly(methoxy di(ethylene glycol)acrylates) **3a**–**3c** prepared via RAFT polymerization were the second class of polymers. For all polymers, a significant influence of the end groups, as well as the molecular weight on the final LCSTs was observed, restricting the investigations to a total of four polymers (the acrylates **3a**, **3b** and **3c**, as well as the poly(oxazoline) **6c**) for the amyloid-fibrillation experiments. Furthermore, the used buffers did show a significant influence on the LCSTs of the polymer in solution, with a significant increase in the case of the poly(acrylates) **3b** and **3c** and a decrease in the case of the poly(oxazoline) **6c**. The polymers (**6c**, **3b**, **3c**) do show a reduction of the lag time of amyloid-aggregation, indicative of an enhanced fibrillation. Although one polymer **3a** did not show an LCST in the used buffer solution, an increase of the lag time of the fibril formation from 18.34–19.33 h was observed.

In summary, several factors can be considered as being influential for the polymer/amyloid aggregation in the current system: (1) enhanced nucleation due to Aβ-cluster formation at the polymer chains at temperatures below the LCST [36,66]; (2) steric shielding of the amyloids as a consequence of polymer adsorption (polymers **3a**, **3b** and **3c**) [15,30,31,32]; (3) enhanced nucleation of amyloid aggregation via the formed polymer particles at temperatures above the LCST (polymer **6c**) [3,27]. Among the discussed possibilities, the enhanced nucleation by all of the used polymers seems the most probable scenario, as in all cases, fibrillation of the amyloids is enhanced, irrespective of their chemical or physical nature. Research is proceeding in this direction, as currently, other mechanisms cannot be ruled out.

## Figures and Tables

**Table 1:** Measured *T*_CP_ for the synthesized poly(methoxy diethylene glycol)acrylates (**3**), poly(2-isopropyl-2-oxazoline) (P*i*PrOx) (**6**–**8**) and P(*i*PrOx-*n*BuOx) (**9**) in sodium borate buffer and in water. For the homopolymers, 1 wt % solutions were used, whereas for the copolymers, 0.25 wt % solutions were used due to poor solubility in water. The samples were heated at a rate of 0.5 K/min. PDI = Polydispersity Index

Polymer	*n*_NMR_^1^ (4)	*n*_NMR_^1^ (5)	*M*_NMR_ (g/mol)	*M*_GPC_ (g/mol)	PDI	*T*_CP_ (°C) (Sodium borate buffer)	*T*_CP_ (°C) (H_2_O)
**3a**	–	–	3,600	2,700	1.1	– ^4,5^	34.4
**3b**	–	–	8,500	6,600	1.2	52.7 ^4^	40.4
**3c**	–	–	14,600	13,700	1.2	49.5 ^4^	45.1
**6a**	1	–	1,600 ^2^	2,600 ^3^	1.2	43.6	47.5
**7**	1	–	2,000 ^2^	3,100 ^3^	1.1	42.6	43.4
**6b**	1	–	4,300 ^2^	4,200 ^3^	1.3	40.1	42.3
**6c**	1	–	5,600 ^2^	11,000 ^3^	2.0	32.2 ^6^ (36.2) ^4^	36.4 ^6^
**6d**	1	–	2,800 ^2^	7,400 ^3^	1.9	31.1/36.5 ^6^	37.8 ^6^
**6e**	1	–	2,300 ^2^	6,200 ^3^	2.0	37.6 ^6^	38.1 ^6^
**8**	1	–	5,200 ^2^	3,000 ^3^	1.3 ^7^	49.8	46.5
**9a**	0.79	0.21	1,700 ^2^	3,600 ^3^	1.3	24.9	27.2
**9b**	0.80	0.20	1,600 ^2^	3,600 ^3^	1.3	24.7	25.7
**9c**	0.61	0.39	1,700 ^2^	4,200 ^3^	1.4	14.2	15.3

^1^ Ratios of the monomers (**4** and **5**) within the copolymers as calculated from ^1^H-NMR; ^2^ the ^1^H-NMR signal from the methylene group next to the alkyne group at δ = 4.05 ppm was used as a reference for polymers **6**, **7** and **9**, whereas for polymer **8**, the signal from the methyl-initiator group at δ = 3.05 ppm was used; ^3^ measured in DMF with polystyrene (*M*_P_ = 1000–115,000 g/mol) as the standard; ^4^ sodium phosphate buffer (pH = 9.2, 150 mmol NaCl) was used instead with a polymer concentration of 230 μmol/L; ^5^ no LCST detectable under the used conditions; ^6^ due to the higher PDI, the drop in transmission is much less pronounced in comparison to the other samples; polymer **6d** exhibits two LCSTs when measured in buffer solution; this might be due to its relatively high polydispersity; ^7^ bimodal distribution according to GPC.

**Table 2:** Measured data for the fibrillation kinetics of the Aβ_40_ fibrillation in the presence of the polymers **3a**, **3b**, **3c** (Fluorescence Protocol II) and **6c** (Fluorescence Protocol I, see Methods) at 37 °C. The characteristic time and lag time vary between the different protocols, as shaking times varied between the two protocols. Shaking increases the probability of molecular contacts, thus enhancing aggregation.

Name	*T*_CP_ (°C)	WT concentration ^1^	*M*n (kDa)	Polymer concentration ^1^	*t*_char_ (h)	*t*_lag_ (h)	Kinetic protocol
Aβ_40_ WT	–	230 μM	4.3	0	27.9 h	18.3 h	II
**3c**	49.5 ^1^	230 μM	14.6	260 μM	18.3 h	15.9 h	II
**3b**	52.7 ^1^	230 μM	8.5	113 μM	12.5 h	9.1 h	II
**3a**	– ^1,2^	230 μM	3.6	196 μM	21.1 h	19.3 h	II
Aβ_40_ WT	–	230 μM	4.3	0	6.4 h	4.9 h	I
**6c**	36.2	230 μM	11.0	230 μM	4.5 h	3.7 h	I

^1^ Cloud points were determined in sodium phosphate buffer (pH = 9.2, 150 mmol NaCl) with a polymer concentration of 230 μmol/L; ^2^ no LCST detectable under the used conditions.

## Supplementary Materials

The following are available online at www.mdpi.com/2073-4360/8/5/178/s1. Figure S1: MALDI of **3a**, Figure S2: ^1^H-NMR spectrum of **3a**, Figure S3: ^1^H-NMR spectrum of propargyl-initiated P*i*PrOx **6a**, Figure S4: ^1^H-NMR spectrum of propargyl-initiated P*i*PrOx **7,** Figure S5: ^1^H-NMR spectrum of methyl-initiated P*i*PrOx **8**, Figure S6: ^1^H-NMR spectrum of P(*n*BuOx-*grad*-*i*PrOx) **9**, Table S1: Characterization of P(*n*BuOx-*grad*-*i*PrOx) copolymers **9**, Table S2: Concentration dependency of the LCST for P*i*PrOx **7**, Figure S7: LCST curves for the P(*n*BuOx-*grad*-*i*PrOx) copolymers **9** (in buffer), Figure S8: Molecular weight dependency of the LCST for poly(methoxy di*(*ethylene glycol)acrylates*)* **3** (in water), Figure S9: Molecular weight dependency of the LCST for poly(methoxy di*(*ethylene glycol)acrylates*)* **3** (in buffer).
