# Supplementary material for: Amyloid Beta Aggregation in the Presence of Temperature-Sensitive Polymers
Source: Polymers (Basel). 2016 May 2;8(5):178. doi: 10.3390/polym8050178 (PMC6432434; doi:10.3390/polym8050178)
Supplement: Supplementary file 1 [file polymers-08-00178-s001.pdf]

Supplementary Materials: Amyloid Beta Aggregation in the Presence of Temperature-Sensitive Polymers

Sebastian Funtan, Zhanna Evgrafova, Juliane Adler, Daniel Huster and Wolfgang H. Binder

1. Characterization of Poly(methoxy di(ethylene glycol)acrylate) (3)

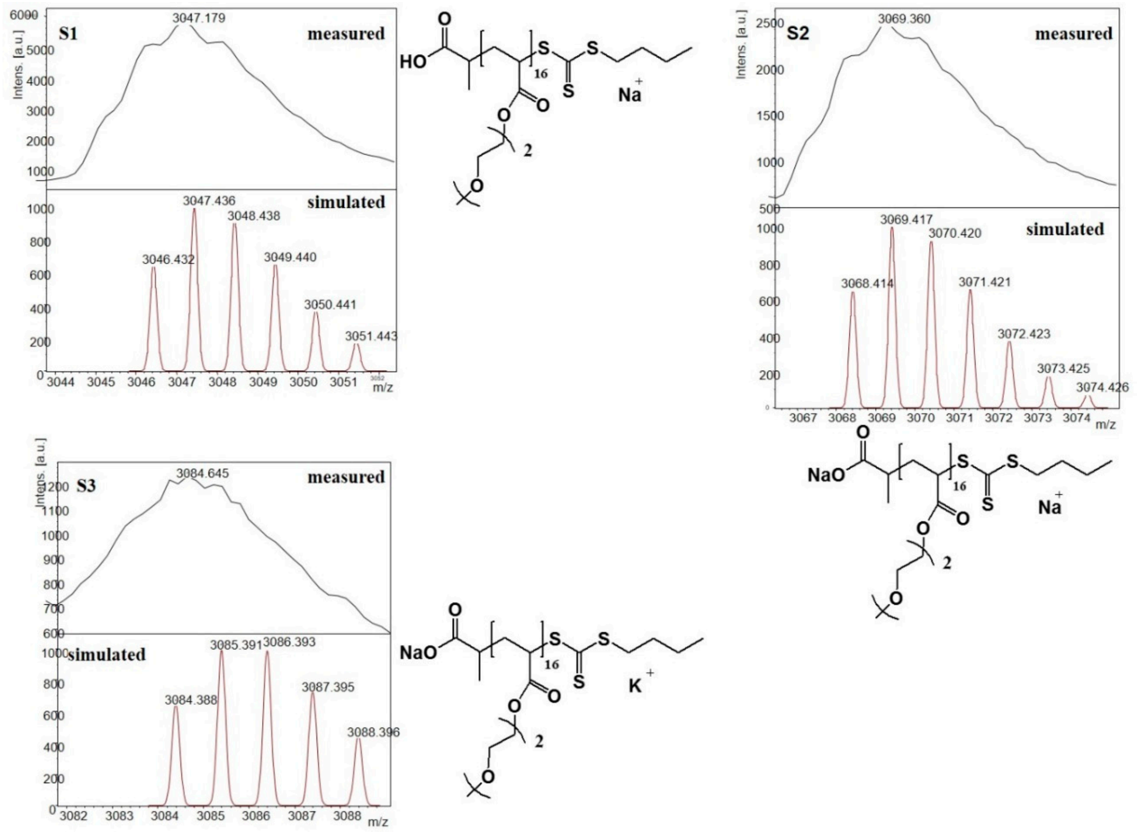

Figure S1. MALDI-TOF spectrum of poly(methoxy di(ethylene glycol)acrylate) (**3a**).

The structure of the obtained poly(methoxy diethylene glycol)acrylate (**3a**) and the presence of both end groups were also verified by ^{1}H-NMR spectroscopy (Figure S2). Thus, an end group methine proton (a) is visible as a multiplet from 4.85 down to 4.82 ppm. The methylene protons (e) of the repeating unit appeared as a broad singlet at 4.19 ppm, while methylene protons (f) + (g) and (h) are present as a multiplet from 3.67 down to 3.62 ppm and a multiplet at 3.53 ppm, respectively. Three methyl protons of the repeating unit (i) were found as a wide singlet at 3.36 ppm. The broad signal at 2.34 ppm can be ascribed to the methine proton (d), and a number of signals from 1.92 down to 1.41 ppm are including protons (c), (k) and (l). The presence of both end groups is verified by the appearance of the protons (b) at 1.15 ppm and (m) at 0.93 ppm. The molecular weight (M_n) of the obtained polymer was assessed as 3600 g/mol using integration values under signal (d) and a molecular weight of the repeating unit equal to 174 g/mol.

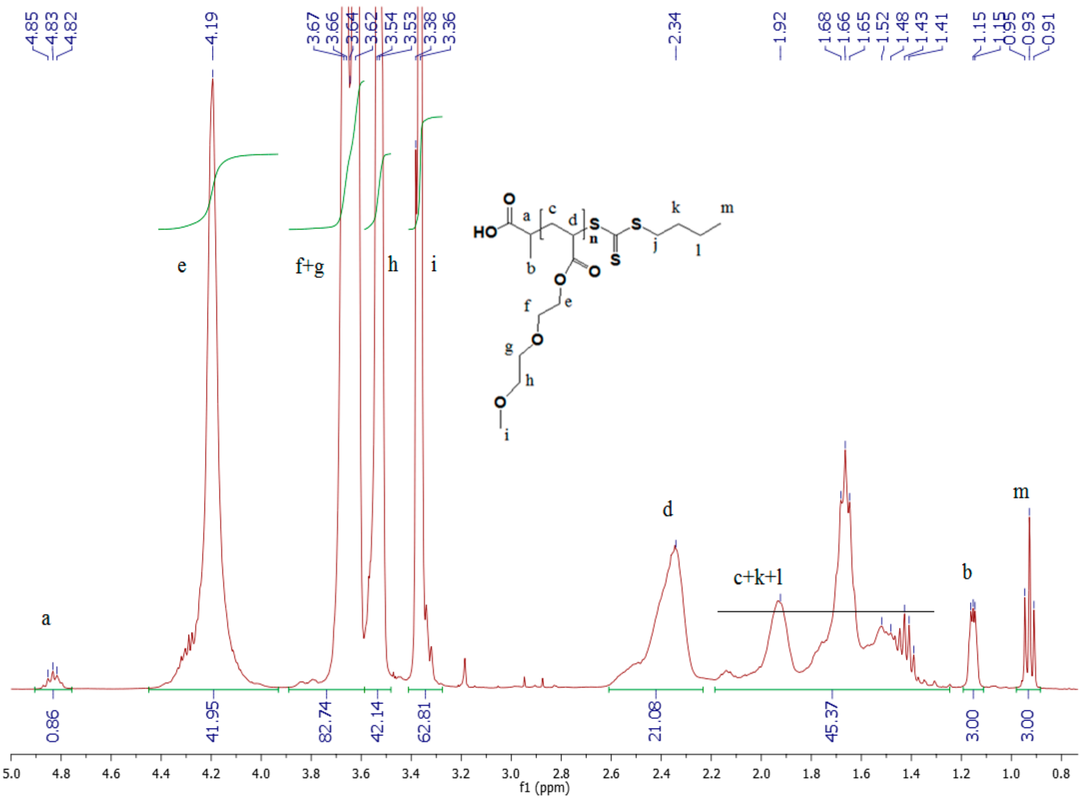

Figure S2. ^{1}H-NMR spectrum of poly(methoxy di(ethylene glycol)acrylate) (**3a**).

2. Oxazoline Syntheses

The procedure for the syntheses of the 2-oxazoline monomers, starting from ethanolamine and the corresponding nitrile, was adopted from Witte and Seeliger [56].

3. Synthesis of 2-Isopropyl-2-oxazoline (4)

To zinc acetate dihydrate (2.2 g, 10 mmol), the isobutyronitrile was added (18.0 mL, 200.0 mmol). Subsequently, the mixture was heated to 130 °C, and the ethanolamine (14.5, 240.0 mmol) was added dropwise to avoid an excessive formation of ammonia. After refluxing for 24 h, the orange solution was distilled under reduced pressure (60 °C, 40 mbar) to yield the pure, colorless product **4**.

*Characterization of Polymer (**4**)*

Properties: colorless, characteristic smelling liquid, boiling point: 41 °C (50 mbar)
Yield: 13.3 mL, 111 mmol (56%)

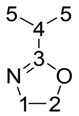

^{1}H-NMR (400 MHz, $CDCl_3$, 27 °C): δ (ppm) = 4.03 (t, 2H, H_2, $^3J_{H,H}$ = 9.5 Hz), 3.62 (t, 2H, H_1, $^3J_{H,H}$ = 9.5 Hz), 2.43–2.31 (m, 1H, H_4), 1.01 (d, 6H, H_5, $^3J_{H,H}$ = 7.0 Hz).

^{13}C-NMR (100 MHz, $CDCl_3$, 27 °C): δ (ppm) = 172.5 (C_3), 67.1 (C_2), 54.1 (C_1), 27.9 (C_4), 19.5 (C_5).

4. Synthesis of 2-*n*-Butyl-oxazoline (5)

A mixture of zinc acetate dihydrate (1.9 g, 8.6 mmol) and valeronitrile (18.0 mL, 173.0 mmol) was heated to 130 °C. Ethanolamine (11.0 mL, 181.0 mmol) was added dropwise to avoid an

excessive formation of ammonia. Consecutively, the mixture was refluxed for 24 h before it was distilled (85 °C, 19 mbar) to obtain the pure, colorless product **5**.

*Characterization of Polymer (**5**)*

Properties: colorless, characteristic smelling liquid, boiling point: 55 °C (15 mbar)
Yield: 9.6 g, 75 mmol (44%)

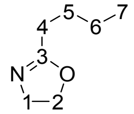

^{1}H-NMR (400 MHz, $CDCl_3$, 27 °C): δ (ppm) = 4.14 (t, 2H, H_2, $^3J_{H,H}$ = 9.4 Hz), 3.75 (t, 2H, H_1, $^3J_{H,H}$ = 9.4 Hz), 2.23–2.18 (m, 2H, H_4), 1.59–1.51 (m, 2H, H_5), 1.36–1.26 (m, 2H, H_6), 0.86 (t, 3H, H_7, $^3J_{H,H}$ = 7.4 Hz).

^{13}C-NMR (100 MHz, $CDCl_3$, 27 °C): δ (ppm) = 168.5 (C_3), 67.0 (C_3), 54.3 (C_1), 28.0 (C_4), 27.6 (C_5), 22.2, (C_6), 13.7 (C_7).

5. Synthesis of Poly(2-isopropyl-2-oxazoline)

The polymerization of the oxazolines was done according to Winnik *et al.* [55], but was carried out with an increased temperature (80 °C) due to lower reaction times.

6. Initiation with Propargyl tosylate (6) + (7)

In the glove box, 2-isopropyl-2-oxazoline (2.00 g, 2.10 mL, 17.63 mmol) (**4**), dry ACN (8.81 mL) and propargyl tosylate were added to a Schlenk tube, which was sealed with a rubber septum afterwards. The mixture was stirred at room temperature for one hour and consecutively for 48 h at 80 °C until gas chromatography (GC) showed complete conversion. The living chain ends were quenched by the addition of water (4 eq.) (polymer **6**) or *N*,*N*-diethylamine (4 eq.) (polymer **7**) and further stirring for 24 h at 60 °C. After evaporation of the solvent, the residue was dissolved in DCM (5.0 mL) and was extracted with water (5 × 30.0 mL). The combined aqueous phases were back extracted using DCM (10 × 30.0 mL). Subsequently, the organic phases were combined and dried over sodium sulfate. After filtration, most of the solvent was removed using a rotary evaporator. The remaining viscous solution was precipitated three times in a cold mixture of diethyl ether/*n*-hexane to obtain the pure polymer. To increase the yield, the solvent was centrifuged after every precipitation.

6.1. Characterization of Polymers (**6**)

Properties: yellowish-orange solid
Yield: 1.65 g, 1.03 mmol (82%)

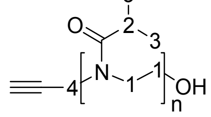

^{1}H-NMR (400 MHz, $CDCl_3$, 27 °C): δ (ppm) = 4.09 (s, 2H, H_4), 3.76–3.26 (m, CH_2 of the repetitive unit), 3.01–2.55 (m, CH of the repetitive unit), 1.09 (s, CH_3 of the repetitive unit).

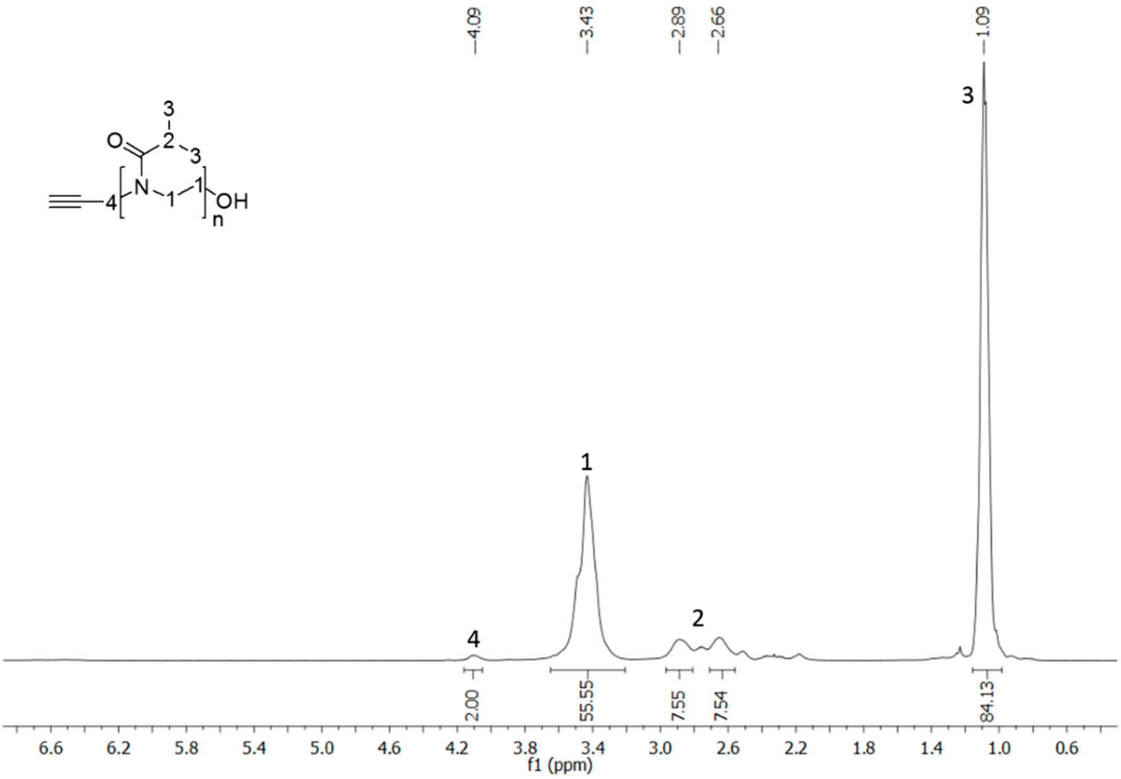

Figure S3. Exemplary ¹H-NMR spectrum from the poly(2-isopropyl-2-oxazoline) homopolymer **6a** that was initiated with propargyl tosylate and quenched with water.

*6.2. Characterization of polymer (**7**)*

Properties: orange solid
Yield: 1.80 g (90%)

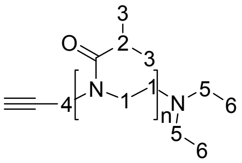

¹H-NMR (400 MHz, $CDCl_3$, 27 °C): δ (ppm) = 4.12 (s, 2H, *H*4), 3.60–3.26 (m, CH_2 of the repetitive unit), 3.00–2.50 (m, C*H* of the repetitive unit), 1.09 (s, CH_3 of the repetitive unit).

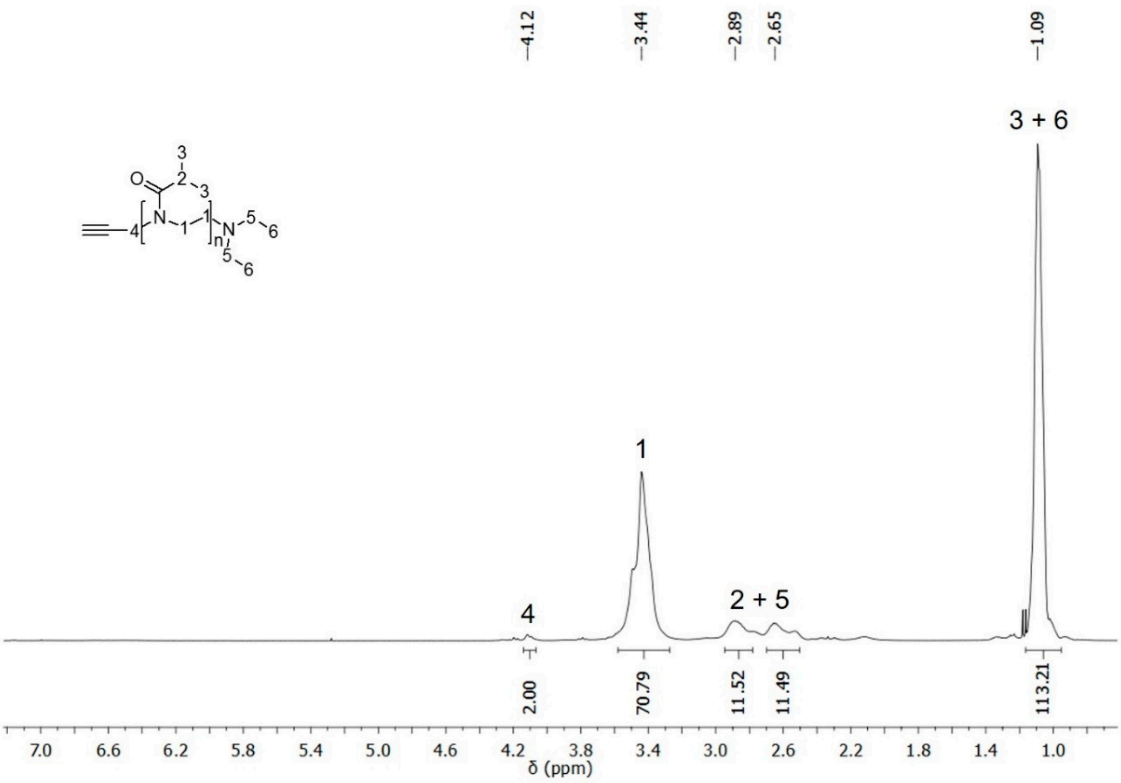

Figure S4. ^{1}H-NMR spectrum from poly(2-isopropyl-2-oxazoline) homopolymer **7** that was initiated with propargyl tosylate and quenched with *N*,*N*-diethylamine.

7. Initiation with Methyl Trifluoromethanesulfonate (8)

The polymerization was carried out as described for the polymerization with propargyl tosylate. 2-Isopropyl-2-oxazoline (0.48 g, 0.50 mL, 4.20 mmol) (**4**), ACN (1.88 mL) and methyl trifluoromethanesulfonate (0.45 M in ACN, 0.21 mL) were mixed in a Schlenk tube and stirred at room temperature for one hour. Subsequently, the mixture was stirred for 48 h at 80 °C. The quenching was done by adding *N*-methylpropargylamine (16.88 μL, 13.82 mg, 0.20 mmol) and stirring for 36 h at 42 °C. The work-up was done as described for the polymerization with propargyl tosylate as the initiator.

Characterization of Polymer (8)

Properties: slightly brownish solid
Yield: 268.0 mg, 51.5 μmol (56%)

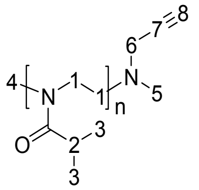

^{1}H-NMR (400 MHz, $CDCl_3$, 27 °C): *δ* (ppm) = 4.09 (s, 2H, *H*$_6$), 3.70–3.30 (m, C*H*$_2$ of the repetitive unit), 3.05 (s, 3H, *H*$_4$), 3.00–2.50 (m, C*H* of the repetitive unit), 1.09 (s, C*H*$_3$ of the repetitive unit).

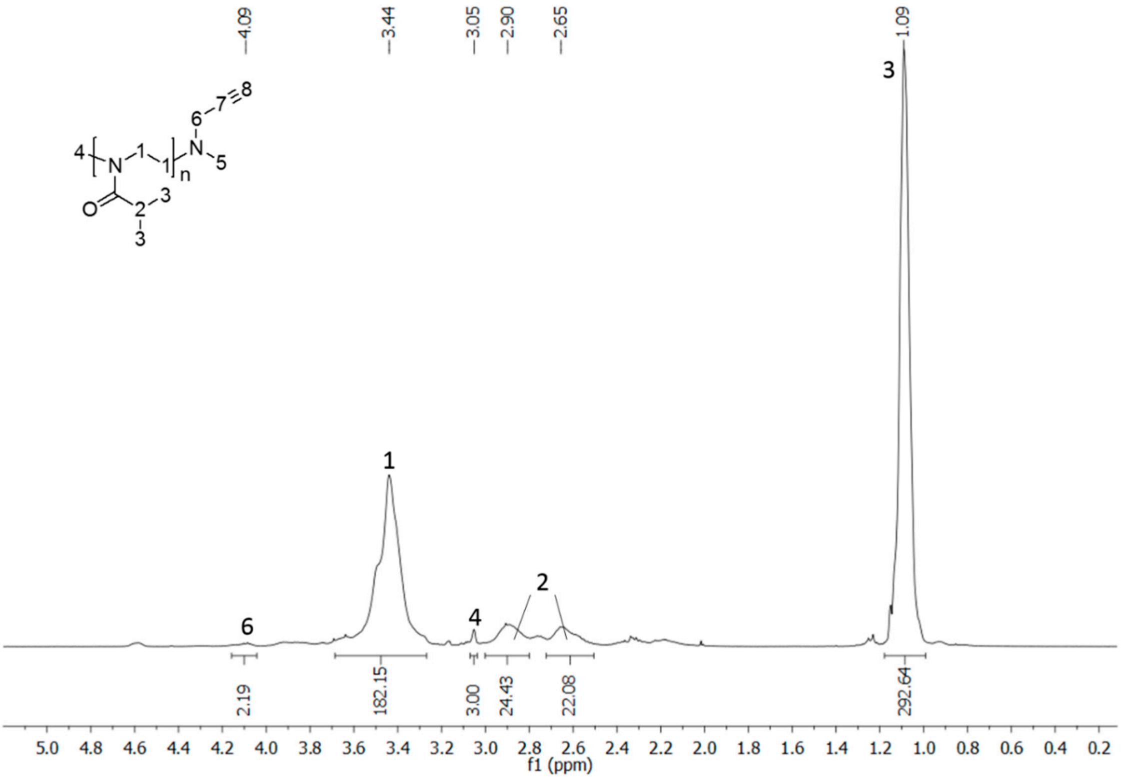

Figure S5. Exemplary ^{1}H-NMR spectrum from the poly(2-isopropyl-2-oxazoline) homopolymer **8** that was initiated with methyl trifluoromethanesulfonate.

8. Synthesis of Poly(2-isopropyl-2-oxazoline-*grad*-2-*n*-butyl-2-oxazoline) copolymer (9)

The procedure was done as described for the homopolymerization of 2-isopropyl-oxazoline. A mixture of 2-isopropyl-2-oxazoline (0.54 mL, 0.51 g, 4.50 mmol) (**4**), 2-*n*-butyl-oxazoline (63.60 mg, 0.50 mmol) (**5**), propargyl tosylate (50.86 µL, 61.80 mg, 0.29 mmol) and ACN (2.50 mL) was stirred for one hour at room temperature in a Schlenk tube. After stirring for 48 h at 80 °C, the reaction was quenched by the addition of water (20.90 µL, 20.90 mg, 1.16 mmol). The reaction was stirred for further 24 h at 60 °C. The work-up was done as described for poly(2-isopropyl-2-oxazoline) [47].

*Characterization of Copolymers (**9**)*

Properties: colorless, slightly yellowish solid
Yield: 527.0 mg, 0.31 mmol (92%)

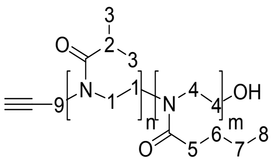

^{1}H-NMR (400 MHz, $CDCl_3$, 27 °C): δ (ppm) = 4.03 (s, 2H, H_9), 3.50–3.25 (m, CH_2 of the repetitive unit, H_1 + H_4), 2.90–2.50 (m, C*H* of the repetitive unit), 2.32–2.15 (m, CH_2 of the repetitive unit, H_5), 1.50 (s, CH_2 of the repetitive unit, H_6), 1.25 (s, CH_2 of the repetitive unit, H_7), 1.02 (s, CH_3 of the repetitive unit, H_3), 0.82 (s, CH_3 of the repetitive unit, H_8).

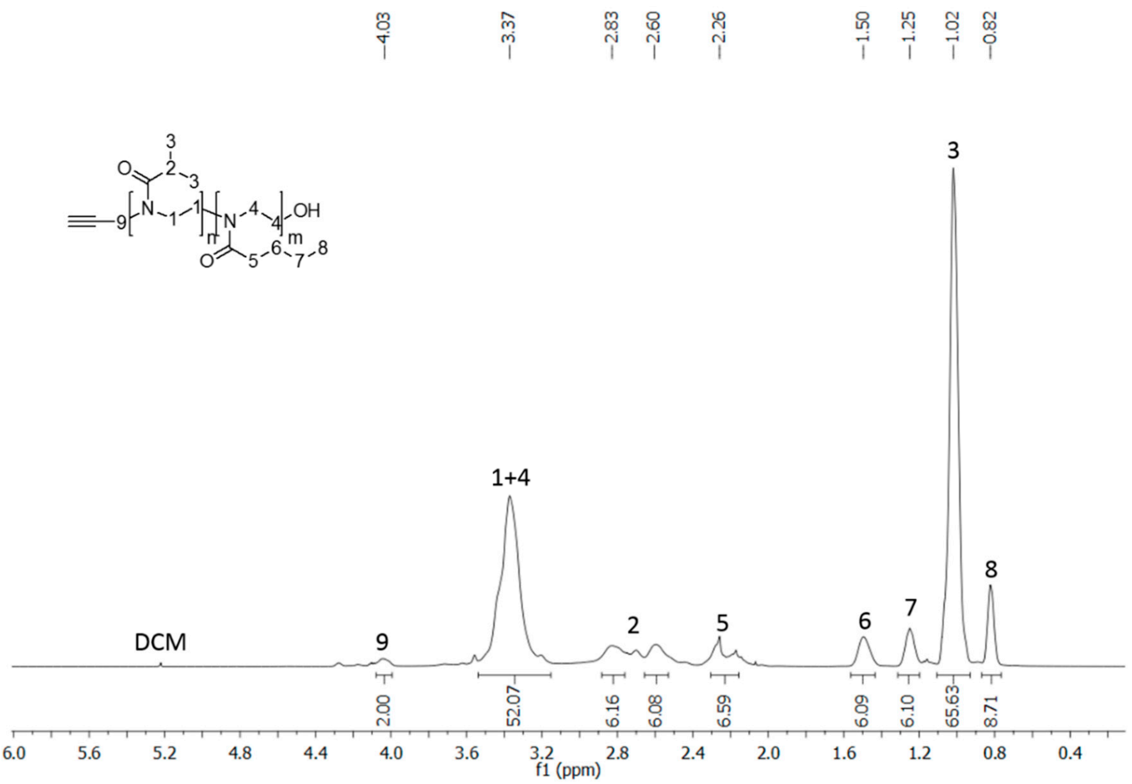

Figure S6. Exemplary ^{1}H-NMR spectrum from the poly(2-isopropyl-2-oxazoline-*grad*-2-*n*-butyl-2-oxazoline) copolymer **9a** that was initiated with propargyl tosylate.

Table S1. Obtained molecular weights, PDIs and compositions for the synthesized poly(2-isopropyl-2-oxazoline-*grad*-2-*n*-butyl-2-oxazoline) copolymers **9** (P(*n*BuOx$_x$*i*PrOx$_y$ where x and y are the numbers of repetitive units within the chain). Polymerizations were carried out in ACN (c = 2 mol/L) at 80 °C with propargyl tosylate as the initiator and water as the quencher.

Entry	$n_{th.}$ [1] (4)	$n_{th.}$ [1] (5)	$M_{theo.}$ (g/mol)	M_{NMR} [2] (g/mol)	M_{GPC} [3] (g/mol)	PDI	n_{NMR} [1] (4)	n_{NMR} [1] (5)	Polymer
9a	0.90	0.10	2,000	1,700	3,600	1.3	0.79	0.21	P(*n*BuOx$_{3}$*i*PrOx$_{11}$)
9b	0.75	0.25	2,040	1,600	3,600	1.3	0.80	0.20	P(*n*BuOx$_{2.6}$*i*PrOx$_{10.4}$)
9c	0.65	0.35	2,060	1,700	4,200	1.4	0.61	0.39	P(*n*BuOx$_{5.5}$*i*POxr$_{8.5}$)

[1] Fraction of the monomer for the copolymer; [2] the ^{1}H-NMR signal from the methylene group next to the alkyne group at δ = 4.05 ppm was used as the reference; [3] measured in DMF with polystyrene (M_P = 1000–115,000 g/mol) as the standard.

9. LCST Measurements for the Poly(2-isopropyl-2-oxazoline) Homopolymer (7)

Table S2. Concentration dependency of the LCST for the P*i*PrOx **7** (3,100 g/mol) in water as 1 wt % solutions.

Concentration	0.25 wt %	0.50 wt %	0.75 wt %	1.00 wt %	1.25 wt %	1.50 wt %
T_{CP} (°C)	48.2	45.4	44.0	43.4	42.3	41.3

10. LCST Measurements for the Poly(2-isopropyl-2-oxazoline-grad-2-*n*-butyl-2-oxazoline) Copolymers

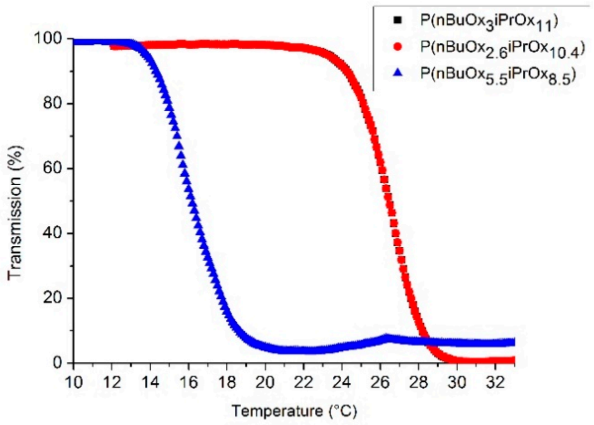

Figure S7. Measured curves for the copolymers **9a**, **9b** and **9c** in sodium borate buffer (50 mmol, pH = 9.0) as 0.25 wt % solution. The presence of the hydrophobic *n*BuOx moieties significantly decreases the LCST. Because of the similar composition of the polymers **6a** (squares) and **6b** (circles), the curves overlap each other.

11. LCST Measurements for the Poly(methoxy diethylene glycol)acrylates

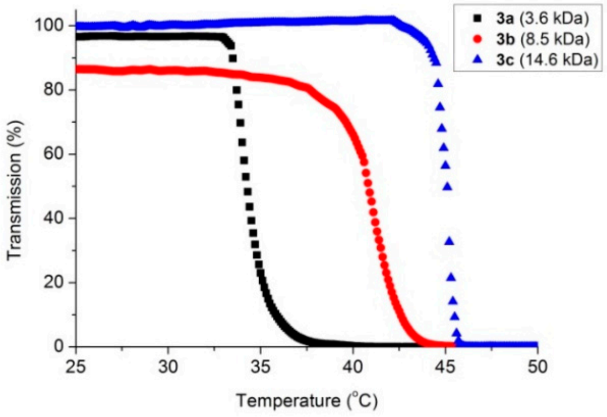

Figure S8. Dependency of the LCST from the molecular weight for 1 wt % solutions of poly(methoxy di(ethylene glycol)acrylates) in H_2O. With an increasing molecular weight, the LCST is increasing.

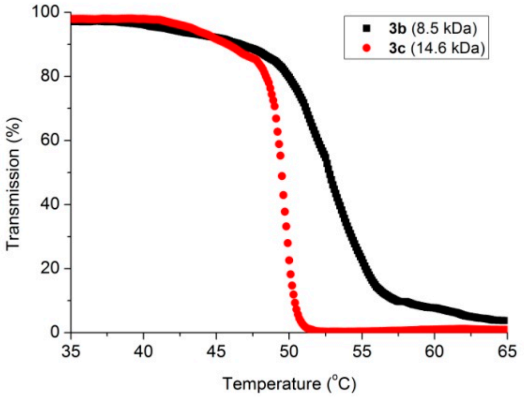

Figure S9. Influence of sodium phosphate (25 mmol/L, pH = 9.2, containing 150 mmol NaCl) on the LCST of poly(methoxy(diethylene glycol)acrylates) **3b** and **3c** (c = 230 µmol/L). The presence of the

salt increases the LCST of the polymer, whereas this affect is much more pronounced for lower molecular weights.

© 2016 by the authors; licensee MDPI, Basel, Switzerland. This article is an open access article distributed under the terms and conditions of the Creative Commons Attribution (CC-BY) license (http://creativecommons.org/licenses/by/4.0/).
